# A never-ending story: The COVID-19 pandemic and the increase of hospital admissions for typical and atypical anorexia nervosa in children, adolescents and young adults in the post-pandemic era in Germany

**DOI:** 10.1192/j.eurpsy.2024.1788

**Published:** 2024-11-15

**Authors:** Beate Herpertz-Dahlmann, Stefan Eckardt, Astrid Dempfle

**Affiliations:** 1 Department of Child and Adolescent Psychiatry, Psychosomatics and Psychotherapy of the RWTH University, Aachen, Germany; 2 Techniker Krankenkasse (Techniker Health Care Service), State Representation North-Rhine-Westphalia, Düsseldorf, Germany; 3 Institute of Medical Informatics and Statistics, Kiel University, Kiel, Germany

**Keywords:** anorexia nervosa, COVID-19, atypical, childhood, adolescence, hospitalization

## Abstract

**Background:**

A large increase in the rate of hospitalizations for adolescents and children with anorexia nervosa (AN) was observed during the coronavirus disease (COVID-19) pandemic. It is still not clear whether this was a temporary effect or whether the increased admission rates persist.

**Methods:**

Data were retrieved from the largest health insurance in Germany comprising 2.5 million children between 9 and 19 y. All patients of this age group with a discharge diagnosis of typical (AN) and atypical AN (AAN) according to the International Classification of Diseases, Tenth Revision (ICD-10), were included. Admission rates per 10,000 person-years were computed separately by sex, age and type of AN for entire years from 2019 to 2022 and the first half of 2023 in relation to the entire number of insured persons of the same sex and age per year.

**Results:**

Two years after the final lockdown admission rates were still significantly higher in adolescent and childhood AN than in the pre-COVID-19 time. While admission rates declined for adolescents in 2023, those for children remained high, with an increase for girls of more than 40% compared with the rate before the pandemic (1.42 (CI 1.26, 1.60); *p* < 0.0001). The highest admission risk for AAN relative to the pre-COVID-19 period was observed in adolescents in the first half of 2023 (1.6; CI 1.34; 1.90; *p* < 0.0001).

**Conclusions:**

Children appear to be especially vulnerable to the pandemic-associated disruptions. Clinicians should try to determine the ongoing effects of the pandemic and support early detection and treatment of AN to prevent its often lifelong consequences.

## Introduction

Eating disorders (EDs), especially anorexia nervosa (AN), are severe mental disorders associated with high personal and societal burdens. During the COVID-19 pandemic, the number of hospitalizations for EDs increased across the Western world, with a particular increase in children and adolescents with AN [1–3]. A number of contributing factors were considered: loss of routine and increased boredom followed by increased activity on social media, especially on those web activities glorifying diets and a slim body image [4]; a more pronounced fear of “fatness” due to a reduction in sports activity [5,6]; and social isolation, especially during COVID-19-associated school closures [7] (for a review, see [1]). However, in spring 2022, COVID-19-associated restrictions were abolished in most Western countries. The majority of children and adolescents returned to normal everyday life.

However, it remains unclear whether the prevalence and hospitalization rate of AN decreased after the end of the restrictions. In the majority of recent studies, data collection ends in the summer of 2022 (for a review, see [8]). Several studies have postulated that the increase in ED symptoms was related to the timing of restrictions (e.g., [1]); others have questioned whether the incidence of EDs would return to baseline after the end of the COVID-19 pandemic. In a very recent report from Canada by Toigo et al. [9] with a data assessment from 2010 to 2022 and an end of documentation as late as April 2023, the highest hospitalization rates were reported in the fiscal years 2021–2022, 2022–2023, and 2020–2021 (in this order). In this study, the expected decrease in admission rates similar to those in the pre-COVID-19 period was not reported; instead, significantly higher rates than before were observed. However, post-COVID-19 epidemiological data from other countries are still lacking [10].

Some authors had already reported a relevant increase in the prevalence of childhood AN in comparison with adult and adolescent AN before the COVID-19 restrictions [11–13] (for a review, see [13]). During the pandemic, the problem of a shift to a greater incidence of AN and utilization rate of health care in younger individuals became more evident. However, very few have explored the increase in hospitalization rates separately for different age groups during and after the COVID-19 pandemic. In a study of patients with AN (mean age 14.63 y) conducted in Israel, the age and sex distributions did not differ between the pre- and peri-COVID-19 periods until spring 2021 [14]. In contrast, Haripersad et al. [15] reported an increase greater than 100% in the rates of admission to a tertiary pediatric hospital for youth aged ≤15 years at the start of the pandemic (from January to May 2020) in Western Australia. Similarly, a very recent Canadian study carried out by Auger et al. [16] reported an increase in the number of 10–14-year-old girls and boys hospitalized for AN during the first wave of the COVID-10 pandemic between the beginning of March 2020 and the end of June 2020, followed by an increase in the number of older adolescents with AN in the second wave (September 2020–March 2021).

Another assumption in the context of the pandemic was a probable shift in the ratio of typical to atypical AN (AAN), which has been investigated in very few studies. Agostino et al. [17] and Haripersad et al. [15] reported similar rates of AN and AAN during the first wave of the COVID-19 pandemic in the spring of 2020, whereas Akgül et al. [18] reported a threefold increase in the rates of AAN in children and adolescents in a Turkish tertiary care center for adolescents during the second wave of the pandemic.

We previously performed a survey on changes in rates of admission for AN in childhood, adolescence and emerging adulthood before and during the COVID-19 pandemic [19]. The results were based on a German nationwide representative population until the end of September 2021, e.g., 3 months after the end of the second and final lockdown. In accordance with other findings, we observed a significant increase in the number of hospitalizations among children and young people (CYP). However, in contrast to findings from the pre-COVID-19 era demonstrating a more pronounced morbidity risk for adolescents/emerging adults [20], in the peri-COVID-19 era, we found an alignment of admission rates between CYP [19]. Interestingly, the increase in hospitalization rates was only due to the increase in admissions for AN, but not for AAN.

The aim of the present follow-up investigation was to explore (1) whether the rates of hospitalization for AN and AAN returned to a pre-COVID state or remained on a high long-term plateau 2 years after the last lockdown; (2) whether admission risks for children and adolescents/emerging adults developed differently during and after the COVID-19 pandemic; and (3) whether there was a change in admission risk for AN compared with that for AAN. Our investigation was conducted in a large nationwide representative sample of 2.5 million CYP between 9 and 19 y of age.

## Methods

### Study design

Data evaluation has been described in detail previously [19]. In short, data were retrieved from the largest statutory health insurance institution in Germany (Verband der Ersatzkassen, VdEK). Note that every citizen in Germany is obliged to be a member of health insurance. In July 2023, the members of this health insurance (VdEK) represented more than one-third of all German inhabitants, with more than 28 million members representing a market share of 38.4% (https://www.vdek.com/presse/daten/b_versicherte.html, assessed May 25^th^, 2024).

Data were anonymously delivered for data analysis to BHD and AD. Data calculation was based on demographic data and discharge diagnoses of inpatients who were members of the “VdEK” from all general, pediatric, psychiatric and child and adolescent psychiatric hospitals in Germany between January 2019 and June 2023, thus covering the whole time span of the pre-COVID-19 period, the peri-COVID-19 period, which included two lockdowns with school closures from March until May 2020 and from November 2020 until May 2021, and the post-COVID-19 period. We defined the child group as children aged 9–14 years and the adolescent/emerging adult group as those aged 15–19 years. These groups were selected according to the data storage system of the health insurance and only included emerging adults till the end of 19. In the whole data basis from 2019 to 2023 only 1–4 patients/year younger than 9 y of age were identified.

### Sample

All patients diagnosed with AN and AAN according to ICD-10 codes (F 50.0 and F 50.1), who were members of the statutory health insurance VdEK, were included. The weight threshold for AN was a BMI at or below the 10^th^ age-adapted percentile until the age of 17 years and according to ICD-10 a BMI less than or equal to 17.5 kg/m^2^ in the 18–19-year old group. According to ICD-10 codes, AAN was defined as the lack of one essential ICD-10 criterion for AN; in nearly all cases, the weight criterion was not met, e.g. the BMI exceeded the threshold of the 10^th^ age-adapted percentile. Thus, AAN defined in our study corresponded almost exactly to the definition of AAN according to the DSM-5. All diagnoses were made by the treating physicians, mostly pediatricians or child and adolescent psychiatrists for the youngest group, and by adult psychiatrists as well as specialists in psychosomatic and internal medicine for the young adult group.

### Data analysis

Admission rates per 10,000 person-years were calculated separately by sex and age group on the basis of hospital admission numbers for entire years from 2019 to 2022 and the first 6 months of 2023, and the number of individuals at risk each year. This was inferred from the total number of individuals per age group insured by VdEK in each year (which was available for both sexes combined) and detailed data on age and sex distributions in Germany by the German National Statistics Office (Statistisches Bundesamt, https://www-genesis.destatis.de/genesis/online) to estimate the number of insured male and female individuals in the groups aged 9–14 years and 15–19 years.

We distinguished between cases, i.e., a single hospitalization record, and patients, i.e., an actual individual with more than one possible hospitalization, to assess readmission rates. Because of the storage system of the health insurance institution, only readmissions during the same year could be evaluated.

The 95% confidence intervals for admission rates were based on Wilson’s approximation [21,22]. Admission rate ratios for comparing rates across years were also based on the yearly rates from 2019 to 2023 and are presented with 95% confidence intervals and *p*-values from chi-square tests. The homogeneity of admission rate ratios between age groups (9–14 versus 15–19 y) was tested using the Mantel–Haenszel test. Data analysis was performed by using the package epiR in R [23].

## Results

### Association of admission rates with age and sex

**Figure 1.**
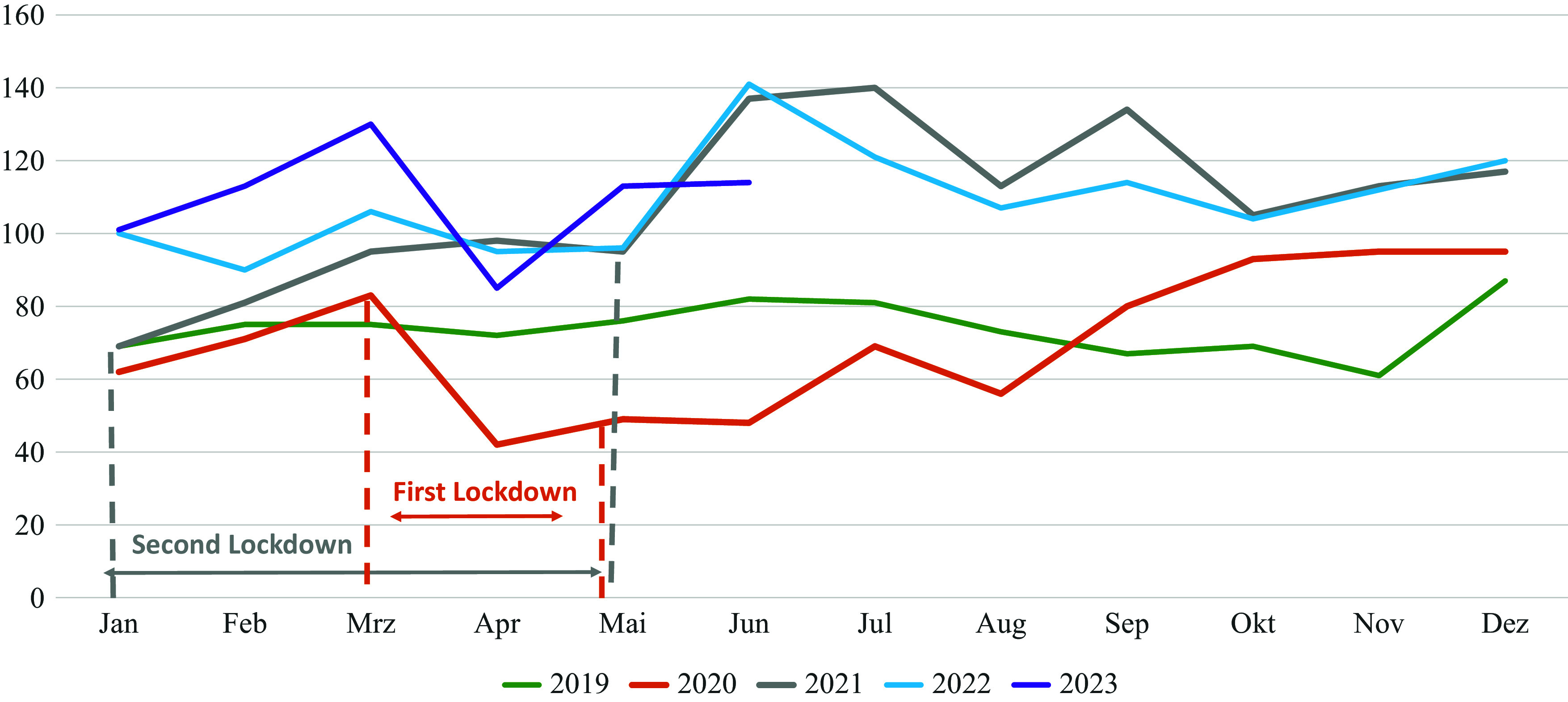
Number of hospital admissions/month in the children’s group from 9 to 14 y. Number of hospital admissions/month in the children’s group from 9 to 14 y. during the entire years 2019–2022 and the first half of 2023 for both sexes with typical and atypical AN according to the ICD-10. Data are based on the largest health insurance set in Germany including all insured persons between 9 and 14 years (about 1.3 million children depending on year).

In **female children** (aged 9–14 years), the consistently highest absolute hospitalization rates from before to after the COVID-19 pandemic (2019–2023) for both AN and AAN were observed for the entire years of 2021 and 2022 as well as for the first 6 months of 2023, e.g., during the height of restrictions and thereafter. In **male** children, the highest absolute admission rate was observed in 2022 but declined to a rate in 2023 as similar to that reported before the pandemic (Fig.1).

As expected, the absolute admission rate was much higher in females than in males. There was no relevant difference in the number of readmissions, i.e., patients treated more than once per year in 2022 (3.8%) compared with 2019 (3.0%).

**Figure 2.**
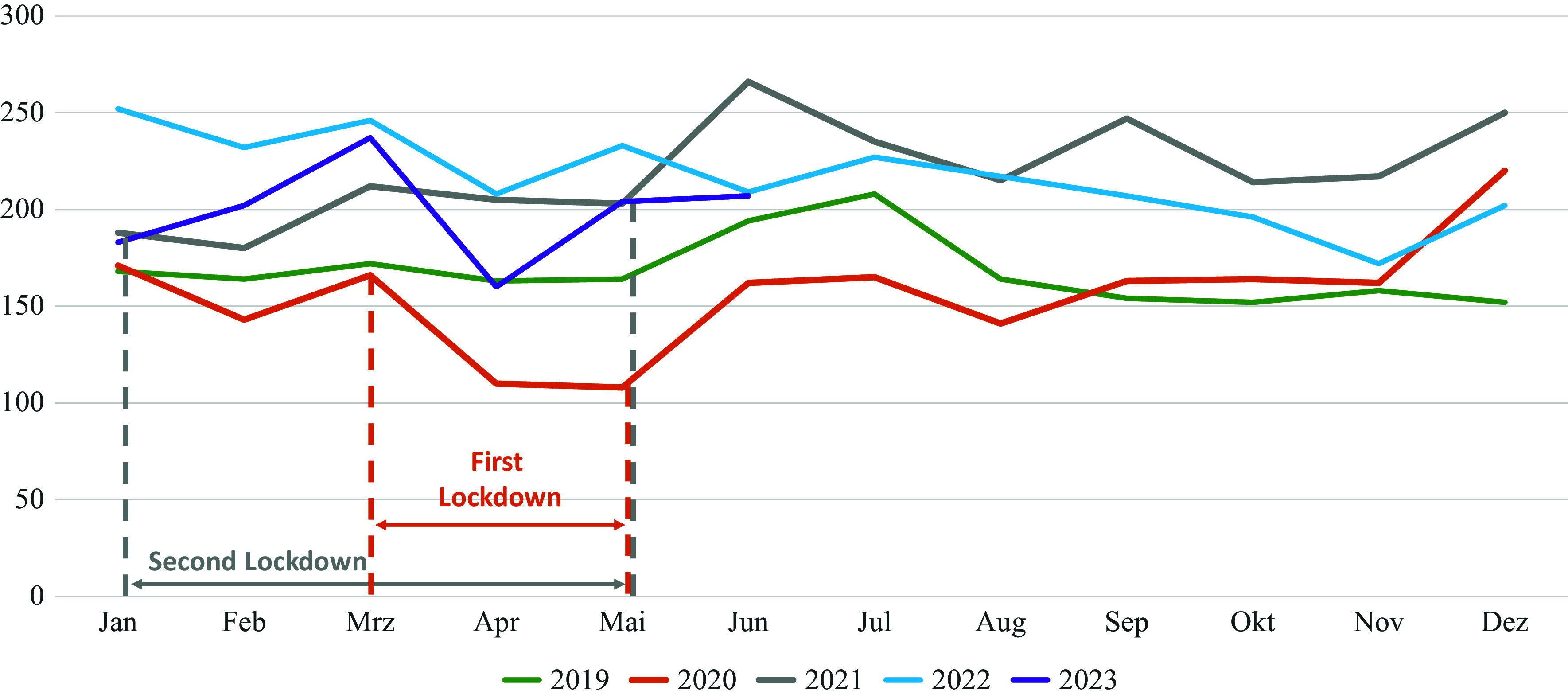
Number of hospital admissions/month in the adolescents/young adult group from 15 to 19 y. Number of hospital admissions/month in the adolescents´/young adult group (15–19 y.) during the entire years 2029–2022 and the first half of 2023 for both sexes with typical and atypical AN according to the ICD-10. Data are based on the largest health insurance set in Germany including all insured persons between 15 and 19 years (about 1.2 million children depending on year).

In **adolescents and emerging adults** (15–19 years), the highest absolute admission rates for girls were observed in 2021, whereas those for boys were observed in 2022. Nonetheless, admission rates for this group were still noticeably higher in the first half of 2023 than in the first half of 2019. (Note, however, that the admission rate for female children was slightly greater than that for female adolescents (p = 0.06) (Fig.2).

Between 2019 and 2022 there was no relevant difference in the number of adolescent/young adult patients readmitted to the hospital in the same year (3.9% versus 4.0%, respectively).

**Table 1a.** Admission rates and admission rate ratios for children (9–14 years) with AN and AAN (collectively) by sex

	2019	2020	2021	2022	2019 (Jan–June)	2023 (Jan–June)	∆ 2020 versus 2019 Admission rate ratio	∆ 2021 versus 2019 Admission rate ratio	∆ 2022 versus 2019 Admission rate ratio	∆ 2023 (Jan–June) versus 2019 (Jan–June) Admission rate ratio
Insured persons (m&f)	13,31,109	13,34,535	13,40,987	13,65,746	13,31,109	13,65,746				
**Females**										
Cases	887	843	1297	1306	449	656				
Admission rate	13.72 (12.85, 14.65)	13.01 (12.16, 13.91)	19.91 (18.86, 21.02)	19.68 (18.65, 20.78)	13.89 (12.67, 15.24)	19.77 (18.32, 21.34)	0.95 (0.86, 1.04)	1.45 (1.33, 1.58)	1.43 (1.32, 1.56)	1.42 (1.26, 1.60)
*p*-Value							0.28	< 0.0001	< 0.0001	< 0.0001
**Males**										
Cases	47	73	70	79	21	26				
Admission rate	0.69 (0.52, 0.91)	1.06 (0.85, 1.34)	1.02 (0.80, 1.28)	1.12 (0.90, 1.40)	0.61 (0.40, 0.94)	0.74 (0.51, 1.09)	1.55 (1.07, 2.24)	1.48 (1.02, 2.14)	1.64 (1.14, 2.35)	1.21 (0.68, 2.15)
*p*-Value							0.023	0.046	0.009	0.62

**Table 1b.** Admission rates and admission rate ratios for adolescents/emerging adults (15–19 years) with AN and AAN (collectively) by sex

	2019	2020	2021	2022	2019 (Jan–June)	2023 (Jan–June)	∆ 2020 versus 2019 Admission rate ratio	∆ 2021 versus 2019 Admission rate ratio	∆ 2022 versus 2019 Admission rate ratio	∆ 2023 (Jan–June) versus 2019 (Jan–June) Admission rate ratio
Insured persons (m&f)	12,50,597	12,14,338	11,81,924	11,73,413	12,50,597	11,73,413				
**Females**										
Cases	2013	1875	2632	2601	1025	1193				
Admission rate	33.37 (31.94, 34.85)	31.93 (30.52, 33.40)	46.03 (44.31, 47.81)	45.92 (44.20, 47.72)	33.98 (31.96, 36.12)	41.77 (39.47, 44.20)	0.96 (0.90, 1.02)	1.38 (1.30, 1.46)	1.38 (1.30, 1.46)	1.24 (1.14, 1.35)
*p*-Value							0.17	< 0.0001	< 0.0001	< 0.0001
**Males**										
Cases	90	81	94	98	47	39				
Admission rate	1.39 (1.13, 1.71)	1.29 (1.04, 1.61)	1.54 (1.26, 1.89)	1.61 (1.32, 1.97)	1.46 (1.10, 1.95)	1.28 (0.94, 1.76)	0.93 (0.69, 1.25)	1.11 (0.83, 1.48)	1.16 (0.87, 1.55)	0.88 (0.58, 1.35)
*p*-Value							0.69	0.53	0.34	0.65

Admission rates (separately for males and females) per 10,000 person-years, together with a 95% confidence interval. Admission rate ratios, together with 95% confidence interval and *p*-value from Chi^2^-test. Total number of insured persons per age group according to health insurance data. According to recent German census data, 51.32% of the population in the age group 0–19 y are male.

### Admission rates in relation to the type of eating disorder (AN or AAN)

Given that the prevalence of AAN is very low among males, we focused only on rates for both AN types among females.

Absolute admission rates for typical AN **in female children** were highest in 2021 and only slightly lower in 2022 and 2023, e.g., in the first half of 2023 still significantly greater than that in the same time span in 2019. In contrast, the rates of admission for AAN in childhood peaked in 2022.

In **female adolescents/emerging adults,** the absolute admission rates for typical AN peaked in 2021 and were only negligibly lower in 2022. In 2023 the numbers were still greater than they were in the first half of 2019. The absolute rate of admission for AAN in adolescents peaked in 2022.

**Table 2a:** Admission rates and admission rate ratios for children (9–14 years) by type of eating disorder (AN or AAN)

	2019	2020	2021	2022	2019 (Jan–June)	2023 (Jan–June)	∆ 2020 versus 2019 Admission rate ratio	∆ 2021 versus 2019 Admission rate ratio	∆ 2022 versus 2019 Admission rate ratio	∆ 2023 (Jan–June) versus 2019 (Jan–June) Admission rate ratio
Insured females	6,46,456	6,48,195	6,51,357	6,63,516	6,46,456	6,63,516				
**typical AN** (F50.0)										
Cases	656	686	1051	1037	343	529				
Admission rate	10.15 (9.40, 10.95)	10.58 (9.82, 11.40)	16.14 (15.19, 17.14)	15.63 (14.71, 16.61)	10.61 (9.55, 11.79)	15.95 (14.64, 17.36)	1.04 (0.94, 1.16)	1.59 (1.44, 1.75)	1.54 (1.40, 1.70)	1.50 (1.31, 1.72)
*p*-Value							0.46	< 0.0001	< 0.0001	< 0.0001
**Atypical AN** (F50.1)										
Cases	231	157	246	269	106	127				
Admission rate	3.57 (3.14, 4.06)	2.42 (2.07, 2.83)	3.78 (3.33, 4.28)	4.05 (3.60, 4.57)	3.28 (2.71, 3.97)	3.83 (3.22, 4.55)	0.68 (0.55, 0.83)	1.06 (0.88, 1.26)	1.13 (0.95, 1.35)	1.17 (0.90, 1.51)
*p*-Value							0.0002	0.58	0.17	0.27

**Table 2b:** Admission rates and admission rate ratios for adolescents/emerging adults (15–19 years) by type of eating disorder (AN or AAN)

	2019	2020	2021	2022	2019 (Jan–June)	2023 (Jan–June)	∆ 2020 versus 2019 Admission rate ratio	∆ 2021 versus 2019 Admission rate ratio	∆ 2022 versus 2019 Admission rate ratio	∆ 2023 (Jan–June) versus 2019 (Jan-June) Admission rate ratio
Insured females	6,03,322	5,87,302	5,71,843	5,66,365	6,03,322	5,66,365				
**typical AN** (F50.0)										
Cases	1560	1452	1988	1927	812	874				
Admission rate	25.86 (24.61, 27.17)	24.72 (23.49, 26.03)	34.76 (33.27, 36.32)	34.02 (32.54, 35.57)	26.92 (25.13, 28.83)	30.86 (28.89, 32.98)	0.96 (0.89, 1.03)	1.34 (1.26, 1.44)	1.32 (1.23, 1.41)	1.15 (1.04, 1.26)
*p*-Value							0.23	< 0.0001	< 0.0001	0.005
**atypical AN** (F50.1)										
Cases	453	423	644	674	213	319				
Admission rate	7.51 (6.85, 8.23)	7.20 (6.55, 7.92)	11.26 (10.43, 12.17)	11.90 (11.04, 12.83)	7.06 (6.17, 8.07)	11.26 (10.10, 12.57)	0.96 (0.84, 1.10)	1.50 (1.33, 1.69)	1.58 (1.41, 1.79)	1.60 (1.34, 1.90)
*p*-Value							0.56	< 0.0001	< 0.0001	< 0.0001

Admission rates (separately for typical and atypical AN) per 10,000 person years, together with a 95% confidence interval. Admission rate ratios, together with 95% confidence interval and *p*-value from Chi^2^-test. Total number of insured persons per age group according to health insurance data. According to recent German census data, 51.32% of the population in the age group 0–19 y are male.

### Admission rate ratios for AN and AAN

In 2023, in female children the admission rate ratio for AN and AAN collectively were similar to those during the COVID-19 pandemic and significantly higher than those before the pandemic (RR: 1.42 [1.26, 1.60]; *p* < 0.0001) (Table 1a). The admission rate ratio specifically for typical AN in girls ≤ 14 y was greater during the entire observation period with the exception of 2019/2020 (see Supplementary Table) than it was in 2019 and still significantly greater in 2023 (RR 1.50 [CI 1.31, 1.72] *p* < 0.0001), while the admission rate ratio for AAN in childhood declined in the first half of 2023 compared with that in 2022 (see Supplementary Table) and was no longer significantly higher than that before the pandemic (RR: 1.17 [0.90, 1.51) *p* = 0.27].

The admission rate ratio for female adolescents/emerging adults with AN and AAN collectively declined in 2023 compared to that during the COVID-19 pandemic but was still significantly higher than that in 2019 (RR 1.24 (1.14, 1.35); *p* < 0.0001).

The highest admission rate ratio for adolescent/emerging adult AAN was found in the first half of 2023 compared with the respective time period in 2019 (RR 1.60 [1.34, 1.90]; *p* < 0.0001). The increase in admission risk for adolescent/emerging adult **AAN** patients was much greater than that for adolescent/emerging adult **AN** patient (*p* = 0.001).

In summary, in the first half of 2023, the admission risk for female children for both types of AN collectively was significantly greater than that for adolescents/emerging adults, but much more so for those with typical AN (*p* = 0.001), whereas for adolescents/emerging adults, the admission risk for AAN was significantly greater than that for children (*p* = 0.049).

## Discussion

To our knowledge, this is the first study to report the prevalence of hospitalizations for childhood and adolescent AN in the pre- and post-COVID-19 periods over four years. Our data are based on a large representative nationwide sample of CYP. The admission risk was separately evaluated for children and adolescents/emerging adults as well as for the type of eating disorder (AN or AAN).

For both age groups, the highest admission rates were observed in 2021 and 2022, e.g., during the peak of the pandemic. While the admission rate ratio in comparison to 2019 was only slightly higher in children than in adolescents/emerging adults until 2022, it obviously declined in the latter group in the first half of 2023, e.g., after the COVID-19 pandemic, whereas in children, the admission rate ratio remained stable at a very high level and was still significantly higher in the post-COVID era than in the pre-COVID-19 time.

The reasons for the shift to an earlier age at hospitalization are not clear. One reason could be a decrease in the age of onset, which had already been described before the COVID-19 period [11,12,24]. However, data concerning a further decrease in the age of onset during the COVID-19 pandemic are scarce. Trafford and coauthors [25] reported that the incidence of EDs (not only AN) in the UK in 13- to 16-year-olds between March 2020 and May 2022 was approximately 42% higher than expected and higher than that for all other age groups. However, the incidence of EDs in boys of the same age was about 16% lower than expected using data from the 10-year period before the COVID-19 pandemic. The latter finding did not correspond to our own study in which admission rates between 2020 and 2022 were also significantly higher in boys up to 14 years, but not in older males. Toigo et al. [9] in Canada also reported a significant decrease in age between 2010 and 2022, with the youngest age at hospitalization during the pandemic. Auger et al. [16] reported an increase in the hospitalization rate for 10–14-year-olds during the first wave of the COVID-19 pandemic (March to August 2020) but not during the second wave. However, the assessment was already finalized in March 2021, which is more than 2 years earlier than in our study. Other recent data are also difficult to compare with our results: in the systematic review and meta-analysis by Madigan et al. [8] higher admission rates were found in adolescents compared to children. However, the huge majority of the studies in this review were only conducted in 2020 and 2021 and the adolescent group included patients ≥12 years, while the definition of adolescence in our investigation comprised patients ≥ 14 years. In our previous study that assessed data until September 2021, the admission risk for children was the same as that for adolescents, but during the post-COVID-19 period it was significantly greater. Adolescence has always been the peak age of onset for AN [20]. Thus, the possible causes underlying the increase in admission risk for children during and after the pandemic are most likely coronavirus-associated changes.

## Psychosocial influences

Rodgers and her group [4] proposed three pathways that might have impacted the mental health of CYP: disruptions of daily structure and confinement, social media and emotional disturbances. Although the pandemic implied a marked disruption for all young people, some restriction-associated measures have probably represented a higher burden for the children’s group than for the adolescents.

In a recent investigation about the effect of school closures on mental health in Germany, younger children struggled most with the disruption in social contacts and interaction. In a sample of more than 1000 11–17-year-olds, the 11-year-olds had the most distinct increase in psychosomatic symptoms as well as in behavioral and emotional problems and the most pronounced decline in quality of life [26]. The same age group reported the strongest reduction in physical activity compared to younger children and adolescents [27]. The authors demonstrated that 11–15-year-olds were more vulnerable to develop anxiety disorder symptoms during pandemic-associated school closures than older pupils [28]. Although social media use is more pronounced in adolescents, the number of German children between 10 and 13 years of age with pathological social media use (according to the ICD-11) increased to about 5% in 2023 (https://www.dak.de/dak/unternehmen/reporte-forschung/dak-studie-mediensucht-2023-24_56536, assessed 25^th^ May). In a study by Rodgers et al. [29] investigating the relationship between social media use, body dissatisfaction and disordered eating in CYP with a mean age of about 12 years a strong association between the frequency of social media use and eating problems was found even among this young age group.

In addition, the younger group seemed to be more affected by family stress, which often increased during the COVID-19 pandemic [26]. In summary, a loss of daily structure and of contact with peers, insufficient social support and family distress may have led children to skip meals and eat less, which are often the first steps in the development of AN.

## Influence of COVID-19-associated weight gain

In Germany [30] as well as in other countries [31], significant weight gain was observed in the young age group during the COVID-19-associated restrictions. The prevalence of obesity increased from 10 to 19% in adolescents, and the increase in the prevalence among children was even greater [30]. This might explain the significant increase in the incidence of AAN, especially in adolescents/emerging adults. According to the DSM-5, AAN is defined as “fulfilling all the criteria for AN, except that despite significant weight loss, the individual’s weight is within or above the normal range.” Several studies reported that individuals with AAN have a higher maximum BMI or a history of obesity compared to patients with AN (for a review, see [32]).

Several young people, especially adolescents who had gained weight during the constraints, most likely took intrusive countermeasures—among others motivated by social media—and lost a considerable amount of weight. This hypothesis could explain the significant increase in AAN in our observation period. In a pre-COVID-19 survey of more than 34,000 children included in the Health Survey for England (HSE), the prevalence of reported weight loss attempts increased significantly from one-fifth of the sample in 1997/1998 to one-fourth in 2015/2016. The greatest change was observed in 13–17-year-old adolescents [33].

To the best of our knowledge, this is the first study to describe a significant increase in the rates of admission for AAN during the COVID-19 pandemic. In our previous study, an increase in the rates of admission for AAN was not observed, similar to other observations in the first wave of the COVID-19 pandemic [17,34]. Most likely, in earlier reports, this problem had not yet evolved because of the short time interval between the onset of symptoms and significant weight loss. In addition, most of the studies did not differentiate between AAN and AN. In contrast to our study, Toigo et al. [9] reported a similar prevalence of AAN in the pre- and post-COVID-19 periods, which might be due to the younger mean age of their sample compared with the adolescent group in our study (from 15 years onwards).

## Limitations

This study has several limitations (1) The diagnoses were made by different clinicians belonging to different specialties, so we could not verify the reliability of the diagnostic criteria. However, the diagnostic criteria for AN are relatively specific and clear and did not change between 2019 and 2023; thus, a systematic trend in diagnostic uncertainty over the observation period seems unlikely. (2) Because of the data system of our health care system, our results are still based on ICD-10, which is no longer the current classification system. However, by using a more strict weight threshold of 17.5 kg/m^2^ our results are more conservative and clearly demonstrate the large increase of AN in CYP during and after the pandemic. (3) Individual patients’ clinical data, such as age, age of onset, BMI, duration of illness, medical state, and number of readmissions beyond the current year, were not available. Thus, we cannot exclude the possibility that children are more likely to be admitted to the hospital than adolescents are, given their disease severity. Again, no systematic trends over time were expected. 3) We could not assess the mean age in the children’s group to obtain a more precise impression of the respective age group, as case data were only available in an aggregated form. 4) Because of the data storage system of the health insurance we could only analyze the data of 18–19 year olds, but not of older patients to cover a larger age span in emerging adulthood.”

In summary, this was not an epidemiological study but rather a description of a clinical population needing hospitalization for AN. The sample covers a large population independent of the medical discipline, the supply structure of hospitals and urban or rural regions.

## Conclusion

To our knowledge, this is the longest observational study of hospitalization rates for AN and AAN in the young population since the beginning of the pandemic. Notably, there was an important increase in AAN diagnoses in adolescents in the post-COVID-19 period following a substantial increase in the prevalence rates of overweight. Thus, we should be aware that an increasing obesity rate might also result in a growing prevalence of AAN, which is likely an underdiagnosed disorder.

While admission rates for adolescents with AN started to decrease in the middle of 2023—although still higher than in the pre-COVID-19 period—this was not the case for childhood AN. The highly increased admission rate for children was not limited to the time of the restrictions but continued well into the postpandemic period. Thus, we conclude that children suffered from pandemic-associated turmoil even more than adolescents did and had more difficulties recovering even after the restrictions had eased. Consequently, we must be aware of the ongoing effects of pandemic-induced alterations and should determine which factors still maintain the increased risk of ED in childhood to develop effective prevention and treatment strategies.

## Supporting information

Herpertz-Dahlmann et al. supplementary material Herpertz-Dahlmann et al. supplementary material

## Data Availability

Restrictions apply to the availability of these data, which were used under license of this study.

## Supplementary material

The supplementary material for this article can be found at http://doi.org/10.1192/j.eurpsy.2024.1788.
